# Work‐Related Asthma From Exposure to Cardboard and Paper Products

**DOI:** 10.1002/ajim.70080

**Published:** 2026-04-06

**Authors:** Mason E. Glanville, Mary Jo Reilly, Kenneth D. Rosenman

**Affiliations:** ^1^ College of Osteopathic Medicine Michigan State University East Lansing Michigan USA; ^2^ Division of Occupational and Environmental Medicine, College of Human Medicine Michigan State University East Lansing Michigan USA

**Keywords:** cardboard, dust, e‐commerce, manufacturing, paper, pulp, shipping, work‐related asthma

## Abstract

**Background:**

We assess the contribution of cardboard dust exposure to the development of work‐related asthma (WRA). Prior studies on paper‐dust‐related breathing problems have focused on exposures in the paper milling and pulp industries. There have been no reports of asthma linked to workplace exposure to cardboard dust.

**Methods:**

From 1988 to 2022, all cases of asthma associated with paper and cardboard dust exposure reported to the Michigan statewide surveillance system were identified. We summarize the characteristics of these workers.

**Results:**

Eight cases of paper‐dust‐related asthma were identified. Seven of the cases were attributed to cardboard dust, and one to paper dust exposure. Half were exposed to cardboard dust while packaging and shipping products in cardboard containers. Five of the cases were new‐onset asthma; the other three were work‐aggravated asthma. No exposures were reported from paper milling or pulp industries.

**Conclusions:**

Cardboard dust was identified as a major contributor to the incidence of asthma associated with paper products. No cases were identified from the paper milling or pulping industry, despite most research studies reporting respiratory disease in that industry. Cardboard dust within manufacturing industries disproportionately contributed to cases of WRA in the state of Michigan, which is not reflected in the medical literature.

## 1 Introduction

Paper dust is included in lists of known workplace asthmagens [1, 2]. Few studies of asthmagens have been conducted outside the paper milling and pulp industries. Increased levels of exposure to paper dust among paper mill workers have been associated with increased airways obstruction on spirometry [3, 4], increased asthma and COPD mortality [5], and increased respiratory symptoms [6]. One study on exposure to paper dust in office settings found an association with an increased risk of adult‐onset asthma [7]. There is also a case report of a worker in a sanitary pad manufacturing facility who had a positive specific inhalation challenge test to cellulose [8]. A case series found positive ELISA responses against corrugated cardboard antigen extracts in workers who packaged photographic film [9]. No published studies were identified that specifically assessed asthma in workers exposed to cardboard dust, despite the global increase in the use of cardboard packaging products related to the expansion of e‐commerce and related shipping industries [10].

Lack of awareness of workplace exposures that cause asthma and failure to report to public health surveillance systems limit the recognition of factors contributing to work‐related asthma. Nevertheless, robust surveillance programs can detect and highlight exposures that may not be widely recognized, enabling investigation and outreach to prevent ongoing worker injury [11]. Here, we report each case of cardboard or paper dust associated asthma reported to the state of Michigan occupational lung disease surveillance system over a 34‐year observation period.

## 2 Methods

We queried the Michigan statewide surveillance system to identify individuals with work‐related asthma linked to paper dust exposure from the period 1988–2022. Cases were included if the individual indicated in their interview, or their healthcare provider indicated in the medical record, that paper or cardboard dust was the agent responsible for their breathing problems.

The state of Michigan surveillance program for asthma and silicosis was initiated in 1988 and later expanded to cover the reporting of all occupational lung disease. It is the oldest continually operated surveillance program for occupational lung disease in the United States [11] and is funded primarily by grants from the National Institute for Occupational Safety and Health (NIOSH), part of the Centers for Disease Control and Prevention (CDC). Pursuant to state law, healthcare providers, hospitals, clinics, and employers are required to report known or suspected occupational diseases to the state within 10 days (Michigan Public Health Code Article 368, Part 56, P.A. 1978, as amended). Reports from all these sources, as well as reports from the state's sole poison center and from the Michigan Workers' Disability Compensation Agency, are received by the Division of Occupational and Environmental Medicine at Michigan State University, which as the bona fide agent of the state, compiles medical records, including pulmonary function tests (PFTs), and conducts standardized telephone interviews of the reported individuals. The standardized telephone interview is completed by staff of the Division of Occupational and Environmental Medicine and includes questions on sex, race, ethnicity, cigarette use, diagnosis of asthma prior to current work where respiratory symptoms are occurring, allergies, family history of asthma, medical history, the history of respiratory symptom onset and persistence, the top two workplace exposures associated with current symptoms, lifetime work and exposure history, asthma medication use, and medical treatment for asthma [11]. Using these medical records and interviews, a physician who is board certified in internal medicine and occupational and environmental medicine uses standardized criteria developed in partnership with NIOSH to categorize each case as either not asthma; new‐onset work‐related, including reactive airways dysfunction syndrome (RADS); or work‐aggravated asthma [12]. See Supporting Information S1 for a flow diagram of the decision logic and case classification for work‐related asthma.

Descriptive statistics are provided on the workers with cardboard or paper exposure work‐related asthma.

This study was reviewed by the Michigan State University Human Subject Review Board and exempted as part of a public health project. Under Michigan law, consent was not required to access patients' health records in the course of the initial public health investigation. Confidentiality and anonymization of patient health data have been maintained in accordance with United States federal law.

## 3 Results

Eight cases attributed to cardboard or paper dust exposure were classified as WRA over the 34‐year period (Table 1). Seven of the cases were associated with exposure to cardboard dust and one with exposure to paper dust. Five of the cases were classified as new‐onset asthma; all five were exposed to cardboard dust. The remaining three cases were classified as work‐aggravated asthma.

**TABLE 1:** Demographic and exposure summary of the eight individuals with work‐related asthma (WRA) following exposure to cardboard or paper dust, Michigan, 1988–2022.

Case	Age	Sex^a^	Race	WRA Classification	Industry	Job Title	Primary Exposure	Latency (years)	Cigarette Smoking
1992	20s	F	White	Aggravated	Manufacturing (telephones)	Assembly	Cardboard	0	Yes
1997	20s	F	White	New Onset	Manufacturing (corrugated box)	Press operator	Cardboard	0	Yes
1999	40s	F	Black	New Onset	Manufacturing (automotive)	Picker/packer	Cardboard	26	No
2000	40s	M	White	Aggravated	Transportation (post office)	Post office automation	Paper	7	No
2004	60s	F	White	New Onset	Manufacturing (corrugated box)	Laborer	Cardboard	2	Yes
2008	50s	M	White	New Onset	Manufacturing (automotive)	Packer	Cardboard	2	No
2014	20s	M	NK	Aggravated	Manufacturing (automotive)	Picker/packer	Cardboard	0	NK
2019	50s	M	White‐Hispanic	New onset	Recycling	Cardboard baler	Cardboard	NK	No

*Note:* NK, not known.

^a^ F, Female; M, Male.

None of the patients worked in a paper mill or paper factory. At the time of the development of their respiratory symptoms, four worked in a manufacturing industry and were exposed to cardboard dust in the process of shipping or receiving manufactured goods in cardboard boxes, two worked in a cardboard box manufacturing facility, one worked at a cardboard recycling facility, and one was exposed to paper dust at a post office. Six reported cardboard dust as the most likely exposure responsible for their symptoms, one reported paper dust, and one reported “dirt,” with cardboard dust being the second most likely exposure. Four were men and four were women. Five workers were White, one worker was Black, one was Hispanic, and the race of one worker was unknown.

Eight of the cases experienced shortness of breath, seven reported chest tightness, five reported cough, and five reported wheezing at work. Seven reported that their respiratory symptoms got better on days when they were away from work, six reported that these symptoms became worse during their shift at work, and five reported that their symptoms worsen throughout the work week.

Two of the eight cases reported a single high‐dose exposure incident at the time of the onset of their breathing problems, while five reported chronic or low‐level exposure over time, and one case had no data on the dose of the initial exposure, but the latency period of their first exposure to their first hospitalization was 18 years. None of the included cases were categorized as RADS. Of the five cases reporting chronic or low‐level constant exposure, the average latency time from the first workplace exposure to paper or cardboard dust to the onset of their first respiratory symptom was 7.4 years, ranging from 0 to 26 years. One patient could not recall the time between first exposure and first symptom, but did endorse being asthma‐free before beginning the job where the exposure took place.

Five of the eight cases presented to an emergency department (ED) at least once for their workplace respiratory symptoms, and two of the patients were each hospitalized twice for asthma (Table 2). Three of the cases were reported by an occupational medicine physician, three by a hospital, one by a pulmonologist, and one by an allergist. The specific workplaces and types of exposures were later clarified during the standardized telephone interview and by review of the medical record including the face page of the medical record that included the employer's name and address. All eight cases were prescribed asthma medication. Only one worker filed a claim for worker's compensation, which was denied. Seven of the eight workers reported smoking cigarettes, with an average exposure of 13.1 ± 20.1 pack‐years. Three were smoking at the time their symptoms developed, while the other four smokers had stopped smoking between 1 and 13 years before the onset of their symptoms.

**TABLE 2:** Medical summary of the eight individuals with work‐related asthma (WRA) following exposure to cardboard or paper dust, Michigan, 1988–2022.

Case	Reported by	Asthma Meds	ED^a^ Visit (#)	Hospital Admission (#)	PFT^b^ Results available	Spirometry	Tested for Hyperreactivity If yes ‐ result
1992	ED	Yes	Yes (NK^c^)	No	Yes	Obstruction	No
1997	Pulmonologist	Yes	Yes (1)	No	Yes	Normal	Yes ‐ positive methacholine challenge
1999	Occupational Physician	Yes	Yes (3)	No	Yes	Normal	No
2000	Allergist	Yes	Yes (1)	No	Yes	Normal	Yes ‐ Negative pre/post bronchodilator spirometry
2004	ED	Yes	Yes (1)	No	No	NK	NK
2008	Occupational Physician	Yes	No	Yes (2)	Yes	Obstruction	Yes‐ Positive pre/post bronchodilator spirometry
2014	Occupational Physician	Yes	No	No	No	NK	NK
2019	Hospital	Yes	No	Yes (2)	No	NK	NK

^a^ ED, Emergency Department (number of visits).

^b^ PFT, Pulmonary function test.

^c^ NK, not known.

None of the cases had PFTs performed in relationship to work. Four were tested for hyperreactivity; three were positive (Table 2).

### 3.1 Case Example

A patient in their 20s, who had been diagnosed with asthma one year before beginning work boxing industrial parts for shipment, experienced multiple acute exacerbations of their asthma three months after beginning work. The patient experienced wheezing, cough, chest tightness, and shortness of breath in association with exposure to a large amount of dust from the use of cardboard boxes. Their symptoms became worse on days when working, worsened over the course of the work week, and improved when away from work. After two months of these symptoms, the patient was prescribed an albuterol inhaler and cough medicine. Spirometry testing revealed a forced expiratory volume in 1 s (FEV_1_) of 51% of predicted, consistent with moderately severe obstruction. Thirteen months after beginning this job, the patient presented to the ED with difficulty breathing. The patient received treatment with a nebulized bronchodilator. The hospital reported the case to the lung disease surveillance system, and the patient was interviewed ten months later. At that time, the patient was still working the same job and experiencing respiratory symptoms at work which were attributed primarily to cardboard dust. The symptoms had lessened since their ED visit, though they were still taking albuterol for respiratory symptoms at work. The patient reported that ten other employees at this facility had respiratory symptoms like these at work, but none of these were reported to the statewide surveillance system. Changes in exposure controls were not reported by this patient following the ED visit for their asthma, and no state OSHA workplace inspection was conducted in this case. The patient was a cigarette smoker since the age of 13 and smoked an average of 15 cigarettes per day. The patient was classified as having work‐aggravated asthma.

## 4 Discussion

From 1988 to 2022, cardboard, not paper, dust exposure, was associated with work‐related asthma in seven of the eight workers reported to a state‐wide respiratory lung surveillance system. These reports are in contrast to the medical literature, where respiratory problems from paper dust exposure have primarily been reported among paper milling and pulp workers. The lack of reports of asthma among paper mill workers is not because of the lack of paper mills in Michigan. Michigan has an important paper production industry. In 2023, there were 190 paper and 67 cardboard manufacturing companies in Michigan employing 8523 and 3974 workers, respectively [13]. Despite the number of companies and employees who work in paper manufacturing, no individuals with work‐related asthma were identified from pulp or paper mills. We cannot explain the lack of reports of cases from paper and pulp mills although it is possible that this is due to differences in access to healthcare and reporting biases between urban and rural areas, or due to differences in workplace exposure controls and hygiene measures in paper mills.

The eight cases identified over the 34‐year observation period suggest that workplace exposure to cardboard or paper dust can lead to acute and chronic respiratory symptoms causing respiratory morbidity. Because work‐related asthma is widely underdiagnosed and underreported [11], we expect the actual impact of cardboard‐ and paper‐induced breathing problems to be greater.

Even though paper dust is a known respiratory irritant, different categories of paper products have rarely been studied as distinct entities. Our findings suggest that cardboard exposure may disproportionately contribute to work‐related asthma among workers exposed to paper dust of any type. Cardboard is an important renewable packaging material, and its production has increased in recent years partly due to the expansion of the e‐commerce and shipping industries [10]. Globally, 407 million metric tons of paper were manufactured in 2014, over half of which was used in packaging [14]. Cardboard is a complex product containing primarily cellulose, hemicellulose, and lignin [15]. Diverse fillers, adhesives, inks, and waterproofing agents may be used depending on the intended application of paper products [16, 17]. Unbleached paper products such as cardboard have a lignin content as high as 2%–10%, versus < 0.5% in bleached paper products like copy paper or hygienic paper [15, 18]. We did not identify any studies which explore the pathophysiologic impact of inhaled lignin, though it is known to incorporate a hydrophobic moiety which likely increases its penetration into the smallest airways [19] More research is needed on exposure to inhalable dusts containing lignin.

In the United States, there is no specific occupational standard for paper dust set by the Occupational Safety and Health Administration (OSHA), but paper dust is instead regulated as a nuisance dust with an allowable total permissible exposure limit (PEL) of 15 mg/m^3^, and 5 mg/m^3^ for the respirable fraction [20]. This level is high enough that there would be visible dust in the air. Paper dust may cause respiratory symptoms through diverse mechanisms. Proposed pathologies include irritant‐driven respiratory dysfunction, sensitizer‐ (IgE) mediated reaction [8], and endotoxin‐mediated reaction, particularly if the paper becomes wet [21]. A study in animals found pure cellulose dust to be biopersistent in the lungs for at least one year, causing a granulomatous reaction and mild interstitial fibrosis [22]. More recent studies found that mice exposed to cellulose nanocrystals had significant lung damage accompanied by elevated neutrophils and eosinophils on bronchoalveolar lavage [23], and increased macrophages, lymphocytes, myeloperoxidase, interleukins of numerous types, G‐CSF, peribranchial and perivascular inflammation, and increased airway responsiveness to methacholine challenge, all of which were worse in females [24]. A consensus statement described repeated irritant asthma as a non‐immunologic form of occupational asthma which is caused by workplace exposure to substances that are capable of inducing inflammation in the airways [25]. The primary exposure in our case series was corrugated board used for packaging, and the airways dysfunction experienced by these workers was most likely irritative rather than sensitizer‐induced. The wide range of manufactured paper products in use today, from traditional brown paper bags to highly engineered oil‐resistant paper food containers, and their diverse array of additives, adhesives, and contaminants equates to the possibility of different exposures for workers throughout many different industries resulting in both sensitizer‐induced and irritant asthma.

One prior study associated each year of exposure to soft paper dust above 5 mg/m^3^ with a 0.87% decreased FEV_1_ [3]. Increased asthma and COPD mortality has also been noted in soft paper mill workers who were exposed to 5 mg/m^3^ for at least five years [5]. However, a prior study found no significant change in spirometry following at least seven years of exposure to soft paper dust ranging from < 1 mg/m^3^ to > 10 mg/m^3^, but did note an increased prevalence of upper and lower airway symptoms including cough and cough with phlegm [26]. Soft paper mills, which produce products such as toilet paper, tissue, and napkins, are likely more dusty than other types of paper mills, and the products contain different additives and softeners such as talc and kaolinite [27] which may confound the interpretation of these exposures.

Other respiratory conditions have been reported with exposure to cardboard and paper dust: a case report of eosinophilic pneumonia in a worker in cardboard manufacturing [28]; an increased incidence of lung cancer in soft paper mill workers [29]; an increased incidence of lung cancer in mill workers which was most pronounced among paperboard millers [30]; and an outbreak of hypersensitivity pneumonitis linked to dry corrugated cardboard [9].

Workers in the rapidly expanding recycling industry also have an increased likelihood of developing WRA from immunogenic microorganisms and endotoxins emanating from contaminated, moist wastepaper [17]. Our study included one worker who developed asthma while working at a cardboard recycling facility, though the exact recycling process at this plant and subsequent mode of exposure were unknown.

Interestingly, inhalable cellulose powder has been proposed as a treatment of allergic rhinitis by creating a gel‐like diffusion barrier over the nasal mucosa. Identical technology has also been explored for use as an excipient for nasally‐insufflated medications [31]. While this technique appears safe and effective in short‐term studies of the upper respiratory tract, the long‐term effects of pulmonary inhaled cellulose should be considered.

Our study has limitations. The information available for inclusion in this study was based on medical records from the reporting hospital and interviews of the cases. The reporting physician made the diagnosis of work‐related asthma (WRA) based on history. No specific antigen challenge testing was performed and no PFTs were performed in relationship to work, and it is possible that some of the subjects could have non‐work‐related asthma or have WRA from other exposures found in these workplaces. However, the final determination for including a case was based on a standardized telephone interview, review of medical records, and review by a board‐certified occupational and environmental medicine physician using standardized criteria (see Supporting Information S1). It is possible that some individuals had irritative symptoms in relation to work but not asthma. None of the workplaces where these cases worked were inspected by OSHA, so we were dependent on patient interviews and workplace characteristics to conclude that paper or cardboard dust was present in the workplace and was the cause of the workers' symptoms. Seven of the cases included in this study had a history of smoking cigarettes, which can confound whether an individual's respiratory symptoms were due to asthma. However, the treating physicians diagnosed all of these individuals with asthma. The diagnosis of asthma and work‐related asthma in the individuals reported in this case series represents the standard of medical care in the United States, and therefore lacks the degree of specificity one would ideally have if PFTs had been performed in relationship to work.

Occupational exposure to inhalable substances is responsible for 16% of all new asthma diagnoses [32], with over 300 agents known to contribute to the burden of this disease [1]. An American College of Chest Physician Consensus Statement concluded that “The substantial prevalence of WRA supports consideration of the diagnosis in all who present with new‐onset or worsening asthma,…” [33] Workplaces and regulatory agencies must recognize the potential of paper fiber dust to cause work‐related lung disease and review workplace exposures, medical surveillance programs, training provided to workers, and workplace air levels to reduce the potential for work‐related health conditions from cardboard and paper dust.

Improved recognition of lung conditions related to workplace exposures such as cardboard and paper dust will lead to more accurate diagnosis of WRA and subsequent intervention by worker health surveillance and enforcement programs, contributing to reduced morbidity from lung function impairment, asthma, airway symptoms, and their associated burdens on the healthcare infrastructure.

## Author Contributions

Mason Glanville was the primary author of this work. He proposed the research question that led to this manuscript, compiled and analyzed the data, and drafted the initial manuscript. Mary Jo Reilly assisted with database research, formatting, and editing of the manuscript. Kenneth Rosenman assisted with the conceptualization and interpretation of the results and provided editing for style and content of the manuscript. All authors approve of the manuscript as submitted and agree to accountability for the accuracy and integrity of its contents.

## Ethics Statement

This study was performed at Michigan State University, East Lansing, Michigan, USA. This study was reviewed by the Michigan State University Human Subjects Review Board and exempted as a public health project. Under Michigan law, consent was not required to access patients' health records in the course of the initial public health investigation. The manuscript contains a case example from a living patient and summary data of patients from a public health surveillance registry. The patients consented to be interviewed in the course of the public health investigation, but their consent was neither required nor obtained for the creation of this report. Confidentiality and anonymization of patient health data have been maintained in accordance with United States federal law.

## Conflicts of Interest

The authors declare no conflicts of interest.

## Supporting information

Supplement 1.
