# Supplementary material for: Work‐Related Asthma From Exposure to Cardboard and Paper Products
Source: Am J Ind Med. 2026 Apr 6;69(6):482–8. doi: 10.1002/ajim.70080 (PMC13157602; doi:10.1002/ajim.70080)
Supplement: Supplementary file 1 — Supplement 1. [file AJIM-69-482-s001.docx]

BOX 3. Work-related asthma (WRA) surveillance case definition for state health

departments

================================================================================================

WRA Surveillance Case Definition for State Health Departments

A. Health-care professional's diagnosis consistent with asthma.*

AND

B. An association between symptoms of asthma and work.+

------------------------------------------------------------------

* Asthma is a chronic condition characterized by inflammation of the tracheobronchial tree

associated with increased airway responsiveness to a variety of stimuli (a). Symptoms of

asthma include episodic wheezing, chest tightness, cough, and dyspnea or recurrent attacks

of bronchitis with cough and sputum production (b). The primary physiologic manifestation

of airways hyperresponsiveness is variable or reversible airflow obstruction. It is commonly

demonstrated by significant changes in the forced expiratory volume in 1 second (FEV1) or

peak expiratory flow rate (PEFR). Airflow changes can occur spontaneously, with treatment,

with a precipitating exposure, or with diagnostic maneuvers such as nonspecific inhalation

challenge.

+ Patterns of association can vary and include a) symptoms of asthma that develop or worsen

after a worker starts a new job or after new materials are introduced on a job (a substantial

period can elapse between initial exposure and development of symptoms), b) symptoms

that develop within minutes of specific activities or exposures at work, c) delayed symptoms

that occur several hours after exposure (e.g., during the evenings of workdays), d) symptoms

that occur less frequently or not at all on days away from work and on vacations, e) symp-

toms that occur more frequently when a worker returns to work, and f) symptoms that are

temporally associated with workplace exposure to an agent with irritant properties. Work-

related changes in medication requirements can accompany these symptom patterns.

Sources:

a. Anonymous. Standards for the diagnosis and care of patients with chronic obstructive pul-

monary disease (COPD) and asthma. This official statement of the American Thoracic

Society was adopted by the ATS Board of Directors, November 1986. Am Rev Respir Dis

1987;136:225-44.

b. Chan-Yeung M, Lam S. Occupational asthma. Am Rev Respir Dis 1986;133:686-703.

BOX 4. Work-related asthma (WRA) surveillance case classification criteria for state

health departments

===============================================================================================================

WRA Surveillance Case Classification Criteria

for State Health Departments

Code Criteria

------------------------------------------------------------------------------------

C1) Increased asthma symptoms or increased use of asthma medication

(upon entering an occupational exposure setting) experienced by

a person with preexisting asthma who was symptomatic or

treated with asthma medication within the 2 years prior to entering

that new occupational setting.

C2) New asthma symptoms that develop within 24 hours after a one-time

high-level inhalation exposure (at work) to an irritant gas, fume, smoke,

or vapor and that persist for at least 3 months.

C3) Workplace exposure to an agent previously associated with

occupational asthma.*

C4) Work-related changes in serially measured forced expiratory volume

in 1 second (FEV1) or peak expiratory flow rate (PEFR).+

C5) Work-related changes in bronchial responsiveness as measured by serial

nonspecific inhalation challenge testing.&

C6) Positive response to specific inhalation challenge testing@ with an agent to

which the patient has been exposed at work.

------------------------------------------------------------------------------------

* Many agents can induce occupational asthma via a specific hypersensitivity mechanism. A comprehen-

sive list of these asthma inducers (a,b,c) is used for this criterion. Known asthma inducers have been

integrated into the Association of Occupational and Environmental Clinics' (AOEC) coding scheme and

have been flagged with the letter "A" (d).

+ Spirometric measurements (e.g., FEV1) can be obtained before and after a person's work shift (i.e.,

cross-shift spirometry). However, many cases of occupational asthma can fail to demonstrate a significant

cross-shift reduction in FEV1, either because of a delayed bronchoconstrictor response or because of

intermittent exposure patterns. Cross-shift spirometry testing on multiple days might help confirm the

association with work. Alternatively, PEFRs can be measured serially throughout the day using a portable

peak flow meter.

& Changes in bronchial responsiveness can be measured by serial inhalation challenge testing with non-

specific agents (e.g., using methacholine or histamine). Evidence of work-relatedness is manifested by

increased bronchial responsiveness (i.e., bronchoconstriction at lower inhaled doses of methacholine or

histamine) following work exposures and decreased or normal bronchial responsiveness after a period

away from work.

@ Specific inhalation challenge testing has distinct objectives, including the following: a) identifying pre-

viously unrecognized causes of occupational asthma, b) confirming a diagnosis of occupational asthma,

and c) identifying the causative agent when more than one allergen is present in the occupational

environment and identification of the causative agent is essential for management. Specific inhalation

challenge testing is potentially dangerous and should be performed by experienced personnel in a

hospital setting where resuscitation facilities are available and frequent observations can be made over

sufficient time to monitor for delayed reactions. Specific inhalation challenge testing is usually not

necessary for clinical diagnosis of occupational asthma.

Sources:

a. Chan-Yeung M, Malo J-L. Compendium 1: table of the major inducers of occupational

asthma. In: Bernstein IL, Chan-Yeung M, Malo J-L, Bernstein DI, eds. Asthma in the work-

place. New York, NY: Marcel Dekker, Inc., 1993:595-623.

b. Chan-Yeung M, Malo J-L. Aetiological agents in occupational asthma. Eur Respir J 1994;

7:346-71.

c. Malo J-L, Chan-Yeung M, Occupational agents. In: Barnes PJ, Grunstein MM, Leff AR,

Woolcock AJ, eds. Asthma. Pennsylvania, PA: Lippincott-Raven Publishers, 1997:1217-44.

d. Hunting KL, McDonald SM. Development of a hierarchical exposure coding system for

clinic-based surveillance of occupational disease and injury. Appl Occup Environ Hyg

1995;10:317-22. The exposure coding scheme is available on the Internet at

<="" font="">
